# Mastering Organoid Growth: A Complete Guide to Overcoming Methodological Challenges

**DOI:** 10.1002/mco2.70571

**Published:** 2026-01-05

**Authors:** Chunbao Jiao, Omer Faruk Karakaya, Neda Dadgar, Chase J. Wehrle, Zahra Massoud, Hanna Hong, Robert L. Fairchild, Nic Leipzig, Federico Aucejo, Wen Wee Ma, Jan Joseph Melenhorst, Sofia Ferreira Gonzales, Andrea Schlegel

**Affiliations:** ^1^ Department of Inflammation and Immunity Cleveland Clinic Research Cleveland Clinic Cleveland Ohio USA; ^2^ Department of Hepatobiliary and Pancreatic Surgery General Surgery Center First Hospital of Jilin University Changchun Jilin China; ^3^ Cleveland Clinic Foundation Enterprise Cancer Institute Translational Hematology & Oncology Research Cleveland Ohio USA; ^4^ Transplantation Center Cleveland Clinic Cleveland Ohio USA; ^5^ CIR Centre for Inflammation Research University of Edinburgh Edinburgh UK; ^6^ Department of Chemical The University of Akron Biomolecular, and Corrosion Engineering Akron Ohio USA; ^7^ Cleveland Clinic Foundation Taussig Cancer Institute Cleveland Ohio USA; ^8^ Cleveland Clinic Foundation Cleveland Clinic Research Center For Immunotherapy and Precision Immuno‐Oncology Cleveland Ohio USA

**Keywords:** cholangiocyte, methodology, organoid, organoid applications

## Abstract

Organoid technology has become among the most popular technologies in recent years, due to their three‐dimensional and physiologically enriching models that closely mimic the structure and function of human organs. Herein, this review details the in‐depth methodology, updated to date, for the efficient cultivation of organoids. Emphasizing liver organoids, both hepatocyte and cholangiocyte derived, and other abdominal organ systems, such as gut, kidney, and pancreas, we explore the technological challenges researchers are facing nowadays, including how to optimize nutrient delivery, maintain cellular diversity, achieve scalability in the organoid culture system, and high‐throughput applications. Addressing those biological and technological complexities, this review aimed at equipping new researchers with practical insights and standardized protocols that will help improve reproducibility and success rates in organoid culture and expand their applications. Furthermore, we discuss current limitations and barriers to clinical translation, highlight key knowledge gaps, and outline emerging innovations, including bioengineering, microfluidic systems, and genetic manipulation, expected to further enhance disease modeling, personalized medicine, and regenerative therapies. Finally, we provide perspective on next‐generation technologies that expedite organoid‐based discovery and development.

## Graphical abstract

Organoids offer transformative 3D models that precisely mimic human organ architecture and function. This review details the latest methodology, emphasizing the cultivation of liver and other abdominal organoids. We analyze technological challenges (e.g., nutrient delivery, scalability, diversity) and provide practical insights for new researchers. Finally, current limitations and emerging bioengineering innovations are highlighted to advance disease modeling and regenerative therapies.

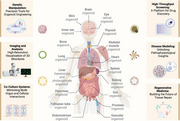

## 1 Introduction

Traditional in vitro models, including two‐dimensional (2D) monolayer cultures and spheroids, have been extensively applied to study cellular behavior, drug metabolism, and toxicity. Cells in 2D cultures grow as flattened monolayers lacking in spatial organization and intercellular communications, significantly limiting their capability for differentiation with specific tissues and physiological responses [1, 2]. These cultures are uniformly exposed to nutrients and drugs, thereby changing their proliferation rates, exhibiting poor polarization, and producing an unfaithful reflection of drug metabolism [3, 4]. Spheroids better preserved natural cell morphology and nutrient gradients and, therefore, can partially mimic the in vivo microenvironment [5, 6, 7]. They exhibit improved cell junctions, drug resistance, and metabolic activity compared with 2D systems but still lack the structural heterogeneity and long‐term stability required for organ‐level functionality [1, 8].

Organoids, compared with other in vitro systems, are three‐dimensional (3D), self‐organized tissue cultures that are derived from stem cells and represent a next‐generation platform capable of self‐organizing into tissue‐specific architectures recapitulating not only cell type composition but also physiological functions, including secretion, transport, and metabolic activities [9, 10], and provide a better predictive model for personalized medicine and precision oncology [11, 12, 13]. Organoid closely replicates the architecture and functionality of real organs and, therefore, is invaluable for disease modeling, drug testing, and regenerative medicine applications [9, 14, 15, 16, 17]. Studies related to this first experienced a major leap in the early 2000s with the advent of advanced stem cell technologies, allowing the generation of more complex and organ‐specific structures based on both pluripotent stem cells and endogenous tissue‐specific progenitor cells [18, 19, 20].

However, huddles still exist in guaranteeing the consistency across different laboratories, regulatory approval for clinical translation, and ethical considerations associated with a source for the stem cells. To researchers who are new to the field, certain essential aspects of organoid research—such as cell sourcing, cell dissociation, culture conditions, growth factor selection, cryopreservation techniques, 3D encapsulation, and quality assessment criteria‐can be highly complicated and challenging to master. These intricacies often create a steep learning curve, hence making it hard for newcomers to establish efficient and reproducible organoid cultures.

This review aims to provide an accessible, beginner‐friendly guide that breaks down critical methodologies into practical steps with a focus on hepatobiliary organoids. Clear instructions and expert insights provided in this work are intended to give the researchers sufficient confidence to overcome such technical challenges, thus allowing for wider adoption and enabling the fullest use of organoid technology for impactful contributions to scientific research. Further, emerging advances regarding next‐generation organoid technologies are discussed, including integrations of bioengineering, high‐throughput automation, and organoid‐based disease modeling, along with their current challenges and future directions that will determine the translational and clinical prospect of organoids in the coming years.

## 2 Materials and Methods

To establish a standardized and reproducible framework for organoid generation, we first detail the essential materials and experimental conditions, including cell sources, media formulations, reagents, and equipment utilized throughout the study.

### 2.1 Cell Source

Two types of stem cells serve as the main cell sources for organoid cultures today.

(1) Pluripotent stem cells, including embryonic stem cells (ESC) and their synthetic induced pluripotent stem cell counterparts (iPSC) and (2) organ‐specific adult stem cells (aSCs). Such aSCs can be isolated from fresh [21] or frozen tissue [22], while iPSCs [23] offer the flexibility to differentiate into various cell types. Each cell source brings distinct advantages and challenges. For liver organoids, fresh tissues provide high aSCs viability and functional fidelity, allowing for precise genetic manipulation and physiological studies [10], that closely mimic in vivo conditions. These features makes fresh tissue‐derived aSCs particularly valuable for personalized medicine [24] and regenerative therapies [21]. However, fresh tissues are often limited in availability, and their use may raise logistic and ethical concerns [25]. Most fresh liver samples come from biopsy, which are typically small in volume requiring specialized handling techniques [26]. While fresh tissue‐based organoid culture demands expertise, it is a viable approach for tissues where biopsy accessibility is not a limited factor [10, 27, 28, 29, 30]. In contrast, frozen tissues offer a practical alternative, maintaining aSCs viability over extended time and allowing flexibility in experimental planning. Rimland et al. successfully employed aSCs from frozen biliary tissues to generate organoids with essential functional characteristics, using the well‐known cell preserving solution “CELLBANKER 2” [31]. The freezing and thawing process can, however, affect aSCs integrity and function necessitating optimal storage conditions. ESCs and iPSCs provide a consistent and scalable source that can be consistently obtained from various well established providers, such as cell banks, research institutions, and biotechnology companies, facilitating high throughput screening and long‐term studies [32, 33]. While aSC‐derived organoids contain only epithelial cells and lack stromal complexity, iPSC‐derived models provide broader differentiation potential yet face challenges in generating diverse stromal lineages, highlighting the need for advanced coculture systems integrating multiple cell types and extracellular matrices (Table 1) [34].

**TABLE 1:** Key differences and challenges in comparing various cell sources for organoid research.

Criteria	Embryonic stem cells (ESCs) [35, 36, 37, 38, 39, 40]	Induced pluripotent stem cells (iPSCs) [36, 38, 39, 40, 41, 42, 43]	Organ‐specific adult stem cells (aSCs) [36, 39, 44, 45]
Source	Derived from the inner cell mass of the 5–7 days old blastocyst	Reprogrammed from adult somatic cells (e.g., skin cells)	Isolated from specific adult tissues or organs
Pluripotency	High pluripotency; can differentiate into all cell types	High pluripotency; similar to ESCs	Limited potency; can differentiate into organ‐specific cell types
Ethical concerns	Significant; involves destruction of embryos	Fewer ethical concerns; derived from adult cells	Minimal; collected from adult tissues
Differentiation control	Well established protocols but can be challenging	Flexible and adaptable; protocols improving continuously	Limited to specific organ types; easier to direct
Tumorigenic potential	Higher risk of teratoma formation	Similar to ESCs; risk of teratoma formation	Lower risk; more stable differentiation
Immunogenicity	Potential for immune rejection unless matched	Lower risk; can be patient specific (autologous)	Minimal risk when using autologous sources
Applications in organoids	Useful for modeling early organ development	Widely used for disease modeling, personalized medicine	Ideal for modeling organ‐specific functions
Time to generate organoids	Can be lengthy; depends on differentiation protocols	Variable; depends on reprogramming and differentiation	Generally faster; already predisposed to organ lineage
Cost	High; requires specialized facilities and ethical oversight	Moderate; initial costs high but cheaper long term	Lower; simpler culture conditions and requirements

### 2.2 Detailed Culture Media Composition and Essential Supplements

The culture medium varies significantly between tissue types, organs, and organoid subtypes. In this article, we will focus on liver organoids (Table 2). The composition of organoid culture media can differ between research groups, based on experimental goal, cell type requirements, and optimization strategy. For example, hepatocyte and biliary epithelial cell organoids have distinct medium formulations (Table 2). The most widely used base medium is advanced DMEM/F‐12 (Thermo Fisher), which provides a balanced nutrient profile supporting cell viability, differentiation, and long‐term culture stability, which being compatible with genetic modification. However, precise supplementation with growth factors is essential for optimizing culture conditions [9, 31, 46, 47].

**TABLE 2:** Overview of media compositions used in liver (cholangiocyte and hepatocyte) organoid cultures.

Author group, year	Reagent	Rotterdam group 2020	Cambridge group 2019 [21, 53]	Cambridge group 2017 [54],2015 [54]	Edinburgh group 2022 [22]	Utrecht group 2013 [55]	Utrecht group 2015 [9]	Japan/Cincinnati group 2021 [56]	Japan/Cincinnati group 2013 [57]	Japan/Cincinnati group 2019 [58]
Organoid type/source of cells		Human extrahepatic and intrahepatic cholangiocyte organoids/primary human tissue	Primary human cholangiocyte organoids/primary tissue	Human cholangiocyte organoids/iPSCs	Human biliary epithelial cell (hBEC) organoids/primary human tissue	Mouse liver organoids (Lgr5+ liver stem cell‐derived organoids)/mouse primary tissue	Human liver organoid/primary human tissue	Human hepato‐biliary pancreatic organoids (HBPOs)/hiPSC	Human iPSC‐derived liver buds/iPSCs	Human liver organoids (HLOs)/iPSCs and ESCs
Cell source detail		Tissue samples collected during liver transplantation (both intrahepatic and extrahepatic regions)	Surgically excised gallbladder or bile duct tissues used to isolate cholangiocytes and generate organoids	Induced pluripotent stem cells differentiated into definitive endoderm, foregut, and cholangiocyte‐like cells	Isolated from discarded donor livers deemed unsuitable for transplantation; cells characterized as CD133+ EpCAM+ CD24+ cholangiocytes with regenerative potential	Derived from bile ducts after liver injury; organoids were expanded from single Lgr5+ cells isolated from damaged liver tissue and differentiated into liver organoids.	Derived from donor liver biopsies; organoids expanded from bile duct cells with bipotent differentiation capacity (hepatocyte and ductal cells)	PSC‐derived gut spheroids were patterned into anterior and posterior domains, and these were fused to generate interconnected hepato‐biliary‐pancreatic organoids.	iPSCs were differentiated into hepatic endoderm cells and cocultured with HUVECs (human umbilical vein endothelial cells) and MSCs (mesenchymal stem cells) to form vascularized liver buds	Induced pluripotent and embryonic stem cells were cocultured to create multicellular liver organoids, including hepatocyte‐, stellate‐, and Kupffer‐like cells for modeling steatohepatitis.
Basal media	Advanced DMEM/F12	1X	—	—	1X	1X	1X	1X**	1X	1X**^, §^
	William's E basal medium	1X^§^	1X	1X^#^	—	—	—	—	—	—
	RPMI 1640	—	—	1X**^, §, §§^	—	—	—	1x*	1X*^,^ **	1X*
	Hepatocyte culture medium (Lonza)	—	—	—	—	—	—	—	—	1X^§§^
Supplements	N2 supplement	1X	—	—	1X	1X	1X	1X**	—	1X**^, §^
	B27 supplement	1X	—	1X	1X	1X	1X	1X**	1X*^,^ **	1X**, ^§^
	ITS+Premix	+*	+	+^#^	—	—	—	—	—	—
	l‐Phospho‐ascorbic acid	0.2 mM*	0.2 mM	0.2 mM^#^	—	—	—	—	—	—
	FBS	—	—	—	—	—	—	0.2%* (only on Day 1) 2%* (only on Day 2)	10%	0.2%* (only on Day 2) 2%* (only on Day 3)
	Insulin	—	—	—	—	—	—	—	1 µg/mL	—
Growth factors	EGF	50 ng/mL (20 ng/mL*)	50 ng/mL	20 ng/mL^#^	50 ng/mL (50 ng/mL*)	50 ng/mL (50 ng/mL*)	50 ng/mL (50 ng/mL*)	—	20 ng/mL	
	FGF‐10	100 ng/mL (50 ng/mL*)	—	50 ng/mL^§§^ (bFGF 80 ng/mL*)	100 ng/mL (100 ng/mL FGF‐19*)	100 ng/mL (100 ng/mL*)	100 ng/mL (100 ng/mL FGF‐19*)	500 ng/mL FGF‐4^§,§§^	10 ng/mL bFGF**	500 ng/mL FGF‐4**
	HGF	25 ng/mL	—	—	25 ng/mL (25 ng/mL*)	50 ng/mL	25 ng/mL (25 ng/mL*)	—	50 ng/mL	10 ng/mL^§§^
	R‐spondin1	10% RSPO1 conditioned media	500 ng/mL	—	500 ng/mL	1 µg/mL or 10% RSPO1 conditioned media	10% RSPO1 conditioned media	—	—	—
	Wnt3a	30% Wnt‐Conditioned media**	—	—	100 ng/mL**	Wnt3a‐conditioned medium**	30% Wnt‐conditioned media**	—	—	—
	Activin‐A	50 ng/mL*	—	100 ng/mL* (50 ng/mL**^,§§^)	—	—	—	100 ng/mL*	100 ng/mL*	100 ng/mL*
	BMP‐7	25 ng/mL^§§^	—	—	25 ng/mL*	—	25 ng/mL^§^	—	—	—
	BMP‐4	—	—	10 ng/mL* (50 ng/mL^§^)	—	—	—	50 ng/mL* (only on Day 0)	20 ng/mL**	50 ng/mL*(only on Day 1)
	Oncostatin M	—	—	—	—	—	—	—	—	20 ng/mL^§§^
	Noggin	25 ng/mL**	—	—	100 ng/mL**	100 ng/mL**	25 ng/mL**	200 ng/mL^§^	—	—
	DKK‐1	—	100 ng/mL	—	—	—	—	—	—	—
Small molecules	A83‐01	5 µM	—	—	5 µM (500 ng/mL*)	50 nM*	5 µM (500 nM*)	—	—	—
	Forskolin	10 µM	—	—	10 µM	—	10 µM	—	—	
	Y27632	10 µM**	10 µM	10 µM**^,§^	10 µM	—	10 µM**	10 µm** (added to medium on Day 7)	—	—
	DAPT	50 µM	—	—	10 µM*	10 nM*	10 µM*	—	—	
	SB‐431542	—	—	10 µM^§^	—	—	—	—	—	—
	LY294002	—	—	10 µM*	—	—	—	—	—	—
	CHIR99021	—	—	3 µM*	—	—	—	2 µM^§^/3 µM^§§^	—	3 µM**
	Retinoic acid	3 µM*	—	3 µM^§§^	—	—	—	—	—	2 µM^§^
	Nicotinamide	10 nM (10 mM*)	10 mM	10 mM#	10 mM	10 mM	10 mM	—	10 mM	—
Buffering agents	HEPES pH7	20 mM*	20 mM	20 mM^#^	10 mM (10 mM*)	—	—	15 mM**	50 mM	—
	Sodium bicarbonate	17 mM*	17 mM	17 mM^#^	—	—	—	—	—	—
	Sodium pyruvate	6.3 mM*	6.3 mM	6.3 mM^#^	—	—	—	—	—	—
Antioxidants and reducing agents	N‐acetylcysteine (NAC)	1.25 mM	—	—	1.25 mM (1 mM*)	1.25 mM	1.25 mM	—	—	—
	2‐Mercaptoethanol	—	—	—	—	—	—	—	50 mM	—
Antibiotics and cell survival agents	Penicillin/streptomycin	100 U/mL/100 g/mL*	100 U/mL/100 µg/mL	100 U/mL/100 µg/mL	100 U/mL/100 µg/mL	—	—	100 U/mL/100 µg/mL**	100 U/mL/100 µg/mL	—
	l‐Glutamine	2 mM*	2 mM	2 mM^#^	2 mM	—	—	2 mM**	2 mM	—
Hormones and differentiation modulators	Gastrin	10 nM	—	—	10 nM (10 nM*)	10 nM	10 nM (10 nM*)	—	—	—
	Dexamethasone	0.1 M*	0.1 µM	0.1 µM^#^	3 µM*	30 µM^§^	30 µM*	—	0.1 mM	0.1 µM^§§^
Metabolic and energy source	d‐Glucose	14 mM*	14 mM	14 mM^#^	—	—	—	—	—	—
Maintenance agent	hES cell cloning recovery solution	–**	—	—	—	—	–**	—	—	—
Notes and comments		*Concentration in cholangiocyte differentiation medium **Present in initiation media for the first 3 days of culture ^§^At fifth day of the cholangiocyte differentiation media, medium was changed to William's E medium ^§§^Expansion media is supplemented with BMP‐7 5 days prior to the initiation of hepatocyte differentiation. Also present in the differentiation media at the same concentration		*Concentration for the differentiation of hPSCs into definitive Endoderm **Concentration for the differentiation of definitive endoderm into foregut progenitors ^§^Concentration for the differentiation of foregut progenitor cells into hepatoblasts ^§§^Concentration for the differentiation of hepatoblasts into cholangiocyte progenitors ^#^Concentration for the differentiation of cholangiocyte progenitors into cholangiocyte organoids	*Concentration in hBEC differentiation medium **Present in expansion media for the first 3 days of culture	*Concentration in hepatocyte differentiation medium **Present in expansion media for the first 4 days of culture ^§^Added to the media during the last 3 days of differentiation for transplantation and in vitro functional studies	*Concentration in hepatocyte differentiation medium **Present in expansion media for the first 3 days of culture ^§^Expansion media is supplemented with BMP‐7 for 5 to 7 days prior to the initiation of differentiation; also present in the differentiation media at the same concentration	*Concentration in definitive endoderm induction medium (Day 0–2) **Concentration in gut growth medium (for 3D spheroid and organoid growth) ^§^Concentration in anterior gut cell induction medium (Day 3–6) (added to gut growth medium) ^§§^Concentration in posterior gut cell induction medium (Day 3–6) (added to gut growth medium) After spheroid and HBPO formation, gut growth medium was used for the rest of the culture period	*Concentration for endodermal differentiation of human iPSCs **Concentration for hepatic specification of iPSCs‐derived endodermal cells	*Concentration in the first 3 days of definitive endoderm induction medium (Day 1–3) **Concentration in Days 4–6 of definitive endoderm induction medium (Day 4–6) ^§^Concentration in human liver organoid induction medium (retinoic acid treatment for 4 days) ^§§^Concentration in hepatocyte culture medium after 4 days of retinoic acid treatment

Abbreviation: EpCAM, epithelial cell adhesion molecule.

The fundamental supplements consist of a pH stable environment, antibiotics, nutritional supplements, and growth factors, which together support regeneration and inhibit differentiation. Typically, the medium includes HEPES for pH stability, penicillin/streptomycin, to prevent contamination and glutamine for enhanced cell viability and growth. Supplements such as B27, N_2_, and nicotinamide can further provide essential nutrients, while N‐acetylcysteine acts as an antioxidant. Growth factors like epidermal growth factor (EGF), fibroblast growth factor 10 (FGF10), and hepatocyte growth factor (HGF) promote cell growth and proteins such as R‐Spondin1 and Wnt support stem cell maintenance by activating Wnt signaling [48]. Inhibitors like A8301 (transforming growth factors‐beta [TGF‐β] inhibitor), Noggin (bone morphogenetic proteins [BMP] inhibitor), and Y27632 (rho‐associated protein kinase [ROCK] inhibitor) help prevent differentiation and apoptosis, maintaining stem cell properties [31, 46, 47, 49]. For tumor organoid cultures, Wnt activators and certain inhibitors such as Noggin are omitted to better model tumor microenvironment and accurately study cancer progression and therapeutic responses [24, 50, 51, 52].

### 2.3 Adaptive Optimization of Growth Factor and Inhibitor Formulations in Organoid Culture

Organoid morphology, proliferation, and differentiation potential are critically dependent on a dynamic balance between stimulatory growth factors and inhibitory signaling molecules. The composition of the culture medium has to be fine‐tuned in most instances using visual morphology and proliferation kinetics. When the organoids exhibit compact morphology with reduced luminal structures, Wnt/β‐catenin signaling is reinforced by supplementing R‐spondin 1 and Wnt3a for the purpose of enhancing the renewal of stem cells and structural stability [9, 31, 54]. On the other hand, excessive budding or cystic expansion may signify the hyperactivation of Wnt signaling and may be counterbalanced by transient withdrawal of R‐spondin or partial reduction of the Wnt3a concentration [59, 60].

Poor proliferation rates, especially for hepatobiliary and pancreatic organoids, are indicative of insufficient stimulation of mitogenic pathways. Increasing the levels of EGF or FGF10 renews proliferative capabilities by driving activity of the ERK and MAPK cascades [31, 57]. In intestinal and gastric organoids, supplementing Nicotinamide or A83‐01, a TGF‐β inhibitor, enhances proliferation and prevents cellular senescence. Prolonged exposure, however, can suppress differentiation and induce abnormal morphology [39, 60]. Cycling supplementation and withdrawal, therefore, is advised for long‐term stability.

Stress induced activation of differentiation pathways may lead to the activation of premature differentiation or apoptosis. Inclusion of Noggin, a BMP inhibitor, and Y‐27632, a ROCK inhibitor, prevents unwanted differentiation and apoptosis by sustaining the epithelial polarity and stemness [31, 49, 61]. In kidney and gastric organoids, when specific lineage differentiation is required, such as nephron progenitors or endocrine cells, partial withdrawal of Noggin or reduced EGF concentration promotes lineage specification [43, 48]. Similarly, in cholangiocyte differentiation, sequential treatment with activin A, retinoic acid (RA), and FGF10 following the initial expansion phase could efficiently induce ductal progenitors, as demonstrated by CK19 and SRY‐box 9 (SOX9) upregulation [18, 54].

Formulation also differs among disease models. For example, in tumor‐derived organoids, Wnt activators, and BMP inhibitors, such as Noggin, are often excluded to preserve better the intrinsic tumor microenvironment and recapitulate cancer‐specific signaling dependencies [45, 50, 51, 52]. In addition, adjusting the concentration of A83‐01 and CHIR99021, a GSK3β inhibitor, allows the controlled induction of differentiation and improves pathological state modeling, including steatosis and fibrosis [9, 31, 62].

Collectively, these adaptive adjustments to culture media provide a flexible and responsive framework that is exploited for optimizing different organoid growth and differentiation. Continuous monitoring of morphological and molecular changes allows for real‐time optimization, ensuring that organoids maintain physiological relevance while responding to experimental objectives (**Table** **3** **)**.

**TABLE 3:** Key reagents and growth factors, and critical considerations in culturing intra‐abdominal organoids.

Organoid type	Liver/hepatobiliary organoids	Gastric organoids	Intestine organoids	Kidney organoids	Pancreas organoids
Key growth factors and reagents	Activin A FGF2/BMP4 Retinoic acid EGF FGF10 Wnt‐3a Noggin R‐spondin BMP7 Gastrin HGF A83‐01(TGF‐β inhibitor Forskolin	Activin A Wnt3a/FGF4/Noggin Retinoic acid EGF Nicotinamide R‐spondin FGF‐10 Gastrin A83‐01 (TGF‐β inhibitor) SB202190 (p38 inhibitor) CHIR99021 (GSK3β inhibitor)	Activin A Wnt3a/FGF4 EGF Noggin Nicotinamide R‐spondin SB202190 (p38 inhibitor) A83‐01 (TGF‐β inhibitor) RANKL	Activin A BMP4 Retinoic acid FGF9 CHIR99021 (GSK3β inhibitor) Noggin VEGF/PDGF	EGF Noggin Nicotinamide R‐spondin FGF10 A83‐01 (TGF‐β inhibitor) CHIR99021 (GSK3β inhibitor)
Key notes and challenges	Endoderm induction: activin A and BMP4 drive definitive endoderm formation, initiating liver organoid generation. FGF2 reinforces this early differentiation stage [31, 57]. Bipotent hepatoblast specification: hepatoblasts, progenitors of hepatocytes and cholangiocytes, emerge under combined effects of FGF10, RA, and activin A, expressing markers such as HNF4A and CK19. These growth factors enable bile duct and hepatocyte lineage development [54, 63]. Organoid expansion: R‐spondin 1 and EGF sustain organoid proliferation. HGF and BMP7 further promote	Posterior foregut specification: activin A initiates definitive endoderm induction, followed by FGF4, Wnt3a, and BMP inhibitors like Noggin to specify posterior foregut fate. This step is critical for forming the stomach's fundic and antral domains [59]. Antral specification: retinoic acid promotes posterior foregut differentiation into antral‐specific gastric organoids. This step ensures proper expression of SOX2 and PDX1, key markers for antral development [59]. Gland and pit formation: the ENRWFG medium (EGF, Noggin, R‐spondin 1, Wnt,	Definitive endoderm formation: activin A is critical for inducing definitive endoderm, the first step in generating intestinal organoids [17]. Hindgut specification and morphogenesis: combined treatment with FGF4 and Wnt3a drives posterior endoderm differentiation into hindgut, while FGF4 alone is sufficient to induce hindgut morphogenesis [65]. Stem cell maintenance: Wnt3a, R‐spondin 1, and EGF are essential for maintaining Lgr5+ intestinal stem cells. R‐spondin 1 acts as a potent Wnt agonist, crucial for stemness and proliferation.	Primitive streak induction: induced using high BMP4 and low activin A or CHIR99021 (Wnt signaling agonist), which specifies posterior primitive streak cells as the source for intermediate mesoderm [71, 72]. Intermediate mesoderm formation: transition to intermediate mesoderm requires FGF9, which mediates renal lineage specification and supports both ureteric bud and metanephric mesenchyme progenitors [72, 73]. 3D organoid culture: self‐organization into nephron structures occurs in 3D cultures, with Matrigel	Adult stem cell‐based organoids: pancreatic organoids are most commonly derived from adult stem cells, particularly ductal progenitors, due to their relatively straightforward culture protocols and rapid expansion capabilities [62]. Progenitor expansion: R‐spondin 1 and EGF are critical for Wnt signaling activation, essential for the expansion of Lgr5+ stem cells from ductal progenitors. Noggin supports the maintenance of these progenitors, enhancing their differentiation capacity [61, 62].
	hepatocyte expansion, with BMP7 enhancing hepatocyte‐specific marker expression and improving differentiation efficiency [9]. Nicotinamide plays a critical role in stabilizing cultures, enhancing initial organoid proliferation, and supporting hepatocyte lineage development [9, 31]. Cholangiocyte differentiation: activin A, retinoic acid, and FGF10 direct differentiation into cholangiocyte progenitors, critical for studying bile duct diseases such as Alagille syndrome and cholangiopathies [54, 64]. 3D culture systems: Matrigel facilitates organoid self‐organization into structures resembling bile ducts and hepatocytes. Wnt3a and R‐spondin are essential for maintaining long‐term stability and genetic integrity during organoid passaging [9, 31, 55]. Challenges in vascularization: The coculture of endothelial (HUVECs) and mesenchymal cells accelerates vascularization and enhances metabolic functionality, mimicking native liver vasculature [31, 57].	FGF10, gastrin) supports self‐organization into gland and pit domains. High EGF levels promote epithelial outgrowth, while lower EGF levels facilitate endocrine cell differentiation [60]. Nicotinamide's role: nicotinamide enhances organoid formation by suppressing sirtuin activity but limits long‐term culture lifespan. It is a facultative component in gastric organoid media [60]. Challenges in long‐term cultures: withdrawal of factors like EGF, Wnt, or R‐spondin rapidly deteriorates cultures. Small‐molecule inhibitors like A83‐01 (TGF‐β inhibitor) and CHIR99021 improve stability and extend culture duration [39, 60]. Endocrine differentiation: low concentrations of EGF allow robust differentiation of gastric	Colon‐specific cultures: in colon organoids, additional Wnt3a supplementation is required compared with small intestine cultures due to the colon's reduced endogenous Wnt production. This ensures proper crypt maintenance and stem cell renewal. Human‐specific challenges: mouse‐derived “minigut” protocols are insufficient for human intestinal organoids, as human epithelial cells require additional small molecules such as nicotinamide, A83‐01, and SB202190 to overcome culture limitations. These additions stabilize cultures and extend organoid lifespan [66, 67, 68, 69]. Epithelial growth: Noggin, in combination with EGF and R‐spondin 1, supports epithelial growth and crypt‐villus structure formation in Matrigel [66, 68]. Endocrine and M cell differentiation: RANKL induces differentiation of M cells, critical for gut immune function, while low EGF levels promote endocrine cell differentiation, including enteroendocrine cells [65, 70]. Long‐term human cultures: nicotinamide, A83‐01, and SB202190 improve the efficiency of human intestinal organoid cultures. These components stabilize cultures, extend their lifespan, and support	or gelbrin ECM facilitating vascular and nephron development. Additional VEGF and PDGF enhance vascularization [74]. Nephron and collecting duct induction: retinoic acid is critical for anterior‐posterior patterning, guiding ureteric bud and nephron progenitor specification. Prolonged CHIR99021 exposure promotes posterior lineage differentiation [72]. Challenges in vascularization: endothelial and mural cell development under static culture is limited. Introducing fluidic shear stress (FSS) in millifluidic chips expands endothelial progenitors (PECAM1+, KDR+) and improves nephron vascularization [74].	Ductal and endocrine differentiation: pancreatic organoids exhibit bi‐potent potential, differentiating into ductal and endocrine lineages when exposed to specific signaling cues. Endocrine differentiation is supported by the inhibition of TGF‐β (via A83‐01) and addition of CHIR99021 [61]. Plasticity and self‐organization: Matrigel provides the extracellular matrix (ECM) environment needed for self‐organization and plasticity. FGF10 enhances early branching morphogenesis and organoid maturation [75]. Long‐term expansion: nicotinamide promotes the stability and long‐term maintenance of organoids, preventing senescence and enhancing proliferation [61]. Challenges in differentiation: spontaneous endocrine
		endocrine cells, such as gastrin and somatostatin‐producing cells, while high EGF levels inhibit this process [59].	differentiation [66]. Challenges in cultures: long‐term cultures require precise control of growth factor gradients, and human samples often face variability in expansion rates compared with murine sample [39].		differentiation is limited in vitro, requiring specific transcription factors (e.g., Pdx1, Neurog3, MafA) for insulin‐expressing β‐cell induction. This highlights the need for fine‐tuning microenvironmental cues [62].
Examples of modeled diseases	Alpha‐1 antitrypsin deficiency [9] Alagille syndrome [9]	Helicobacter pylori Infection: human stomach organoids were infected to study bacterial pathogenesis [60].	Cystic fibrosis: restored swelling response with CFTR correctors in intestinal organoids [76]	Nephrotoxicity: cisplatin toxicity studies on renal structures [72].	PDAC [75]

Abbreviations: Lgr5, leucine‐rich repeat‐containing G‐protein coupled receptor 5; PDAC, pancreatic ductal adenocarcinoma; PDX1, pancreatic and duodenal homeobox 1.

### 2.4 Extracellular Matrix

In contrast to traditional cell culture systems, organoid cultures require an extracellular matrix (ECM) that offers structural support by providing a complex mixture of proteins and growth factors [14, 77, 78]. This support is essential for maintaining the 3D organoid architecture, which is critical to preserve and assess their function and differentiation [79, 80, 81, 82, 83]. Most organoid culture protocols utilize Matrigel as the standard ECM [78, 84], which is a gelatinous protein mixture secreted by Engelbreth‐Holm‐Swarm mouse sarcoma cells, a tumor rich in extracellular matrix proteins [85, 86]. Matrigel primarily consists of laminin, collagen IV, entactin, and heparan sulfate proteoglycans, and various growth factors including EGF, insulin‐like growth factor, FGF, and TGF‐β. The growth factor reduced Matrigel is a version of Matrigel that has been modified to reduce abundance levels of these growth factors [87, 88], which minimizes exogenous signaling influences, making it better suited for controlled studies (Table 3) [88, 89, 90, 91].However, despite its widespread use, Matrigel has notable limitations, particularly for clinical applications [77]. Its murine tumor origin and high batch‐to‐batch variability (up to 50%), compromise the reproducibility of experiments and its potential for regulatory approval [89, 90]. Due to its somewhat undefined composition and animal origin, Matrigel cannot comply with good manufacturing practices (GMP), lacking United States Food and Drug Administration (US FDA) approval, making it currently unsuitable for clinical translation [92].

To address these limitations, several synthetic and natural biomaterials have been developed as alternatives. Cellulose nanofibril (CNF) hydrogels have emerged as a promising alternative for clinical‐grade scaffolds. With defined chemistry from plant origins, CNF hydrogels show mechanical properties that support the differentiation of liver organoids and in principle meet the US FDA and GMP requirements [77]. With well‐defined mechanical properties in the ideal liver tissue stiffness (0.2‐1 kPa) and a shear thinning behavior [92], CNF offers tunable material properties and compatibility with bioprinting making it a viable candidate for therapeutic applications integrating organoid technologies [85, 93, 94], including intestinal injury repair [95]. Similarly, other biomaterials are being explored to replace Matrigel in organoid culture. For instance, enzymatically crosslinked polyethylene glycol (PEG)‐based hydrogel modified with laminin or Arg‐Gly‐Asp (RGD) peptides [96] as well as a novel hydrogel based on polyisocyanopeptides (PIC) and laminin‐111 [89], these materials are chemically controlled, GMP‐compliant, reproducible, and modular [97]. Ligand density, degradability and stiffness can be independently adjusted to mimic liver‐specific microenvironments or to guide lineage specification, for example, enabling chemically defined expansion of human bipotent hepatic progenitors in PEG–RGD matrices [98]. Their tunability also opens the door to more advanced multicellular systems, including liver‐on‐chip designs and 3D bioprinting constructs [97]. Composite hydrogels that combine synthetic control with liver‐specific ECM components represent an emerging hybrid solution that maximizes both bioactivity and translational readiness [99]. Other alternatives, like decellularized liver matrix [100], especially those sourced from human or porcine liver, preserve native ECM architecture and bioactive cues that promote functional organization, such as improved bile duct like morphology in cholangiocyte organoids [101]. Nonetheless, donor dependent variability and the inability to precisely tune mechanical or biochemical properties (adhesion/diffusion) remain limitations.

Taken together, engineering improved ECM platforms remain one of the most important requirements to unlock the full potential of liver organoids in regenerative medicine. Transitioning from Matrigel to well defined, scalable and tissue tailored matrices will not only enhance physiological relevance, including zonation, metabolic activity, and ductal function, but also enable safe and standardized manufacturing routes for clinical applications (Table 4).

**TABLE 4:** Extracellular matrix scaffolds used in organoid culture systems.

ECM type	Source	Structure/composition	Advantages	Limitations	Example applications
Matrigel/BME	Animal‐derived (mouse sarcoma; Engelbreth–Holm–Swarm)	Basement membrane extract containing laminin, collagen IV, and growth factors	Excellent organoid formation Supports self‐organization and long‐term culture	Animal‐derived; batch variability Not suitable for clinical translation	Human branching cholangiocyte organoids [7]
Collagen	Animal derived	Major natural ECM fiber providing 3D structural support	Simple composition Tunable stiffness Widely available Low immunogenicity (biocompatible)	Limited biochemical cues Low mechanical strength	Intestinal organoids self‐organize into macroscopic tubes in floating collagen gels [8]
Hyaluronic acid (HA)	Natural polymer	Hydrated GAG network found in soft tissues	Biomimetic softness and good diffusion Customizable	Weak cell adhesion unless modified Rapid degradability	Islet organoids with promising therapeutic effect by combining pancreatic ECM with HA [9]
PEG	Fully synthetic	Chemically defined polymer, bioactive peptides can be added	Fully controlled and modifiable composition Reproducible GMP friendly	Requires functionalization to support robust organoid growth	Human liver organoids, efficiently differentiated into hepatocyte‐like cells [3]
Decellularized ECM	Human or animal organ‐derived	Organ‐derived ECM with preserved proteins and mechanical cues	Closest to native biology; enhances tissue‐like behavior	Variable quality Expensive Limited scalability	Intrahepatic cholangiocyte organoids cultured in human decellularized liver ECM hydrogels [4]
Composite hydrogels	Hybrid; combination or natural and synthetic components	Combines bioactivity and mechanical tunability	Best balance between growth and differentiation	Formulation optimization needed Higher cost	Liver organoids consisting of hepatocyte‐like and cholangiocyte‐like cells [1]

### 2.5 Equipment

For successful organoid culture, researchers must use specialized equipment to ensure sterility, proper cell isolation, and optimal growth conditions. All organoid culture work must be conducted in a Class II laminar flow biosafety cabinet to maintain a sterile environment and ensure biosafety, as most samples originate from human sources [102]. A temperature‐controlled shaker set to 37°C [47] or a GentleMACS Dissociator [9] is required for mechanical dissociation and enzymatic digestion during cell isolation from tissue. A refrigerated centrifuge is essential for various steps in organoid preparation, including cell pelleting and washing [71]. To maintain optimal growth conditions, such as temperature, humidity, and CO_2_ levels, organoids must be cultured in a cell culture incubator set to 37°C with 5% CO_2_ [67]. An inverted microscope is necessary for observing and monitoring organoid growth and morphology [9], while a live cell imaging system such as an IncuCyte or fluorescent microscope can provide critical insights in to proliferation, morphology, and viability.

Cryopreservation equipment, including a controlled‐rate freezer and liquid nitrogen storage tank, is essential for the long‐term preservation of organoids [103]. Additionally, commonly used laboratory supplies such as a water bath, sterile tips, culture plates, centrifuge tubes, cryovials, and filters are necessary for routine organoid culture and maintenance.

## 3 Stepwise Approach to Organoid Culture and Analysis

Building on the defined materials and culture components described above, this section provides a comprehensive stepwise framework for organoid generation and evaluation, encompassing tissue dissociation and cell isolation, culture initiation, maintenance, expansion, differentiation, and maturation. It further details molecular and functional assessment methods, including marker‐based analyses, viability assays, and cryopreservation strategies, to ensure reproducibility and long‐term preservation of organoid systems.

### 3.1 Isolation of Cells From Different Tissues

#### 3.1.1 Materials

The section lists the required solutions and materials for cell isolation from tissue samples.

*Enzyme solution*: Collagenase IV (Sigma‐Aldrich), Dispase II (Sigma‐Aldrich), TrypLE Express (Gibco) for enzymatic dissociation of epithelial tissues.

*Buffers and media*: DMEM/F12 (Gibco), phosphate‐buffered saline (PBS; Gibco), Krebs Ringer buffer [104].

*Strainers*: 70 µm nylon cell strainers (Falcon)

*Tissue culture plates*: Six‐well or 24‐well plates (Corning), ultra‐low attachment plates for suspension cultures when required.

*Matrigel*: Growth factor‐reduced Matrigel (Corning), culture factors mix (Table 2)

*Additional supplies*: Sterile pipette tips, centrifuge tubes, low retention pipette tips for handling viscous reagents, and cell counting slides or automated counters for viability assessment.

#### 3.1.2 Method

**FIGURE 1:**
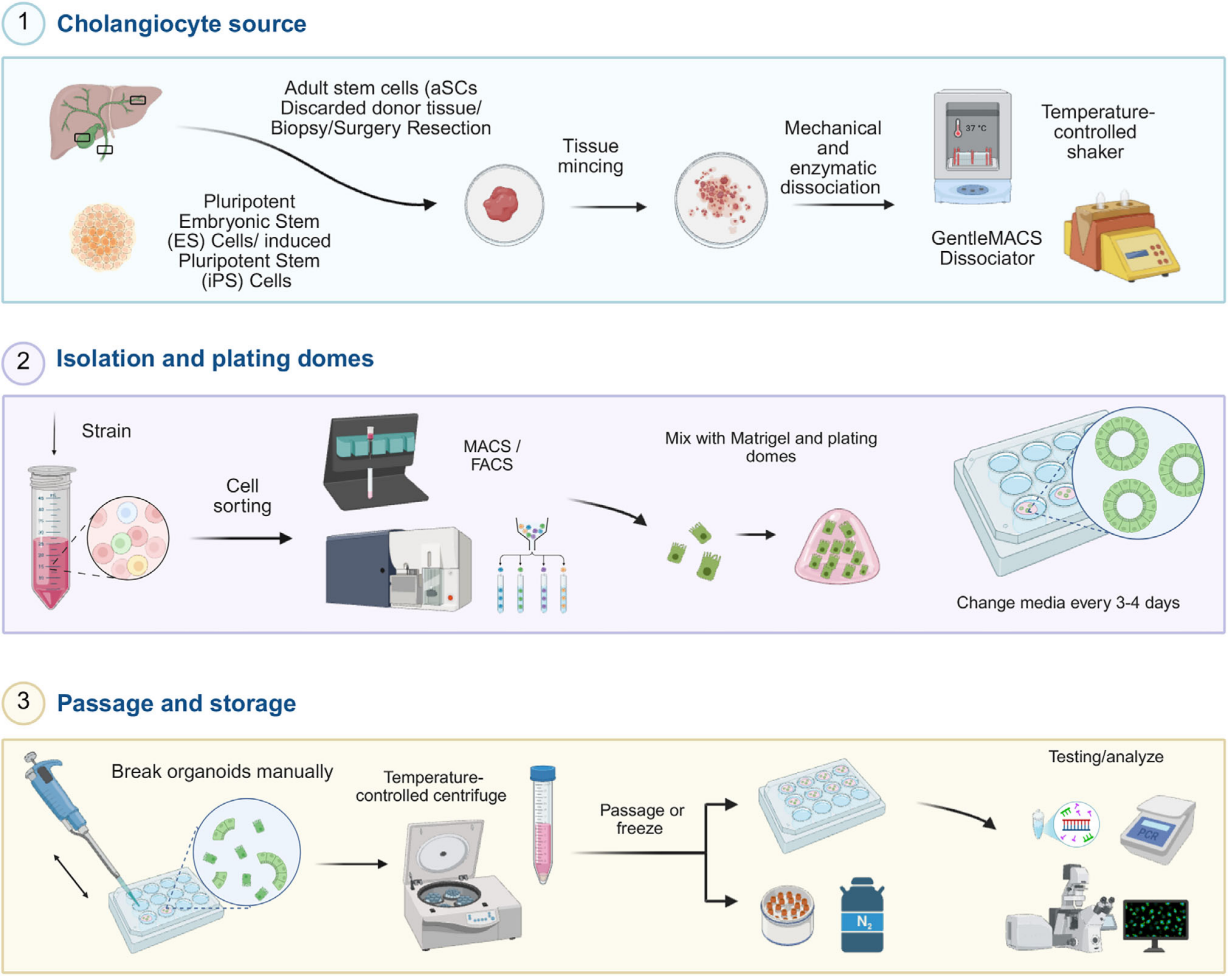
Cholangiocyte organoid workflow: from tissue source to long‐term storage. Cholangiocyte source: Cholangiocytes are obtained from ESCs, iPSCs, or aSCs. For tissue‐derived sources, tissue samples are minced and dissociated enzymatically and mechanically using a temperature‐controlled shaker or a MACS dissociator. Isolation and plating: Dissociated cells are filtered through a 70 µm strainer to remove debris and subsequently sorted via MACS or FACS. The sorted cells are embedded in Matrigel and plated in dome structures, with media frequently changed to support growth and viability. Passage and storage: Organoid domes are manually disrupted, centrifuged, and either replated for further expansion or cryopreserved for long‐term storage in liquid nitrogen. Organoids can later be thawed for downstream applications, including experimental testing. Figure was created using BioRender.

a) *Tissue sampling and storage*: Collect the liver tissue samples from donor organs or tissue resections or biopsies in sterile PBS/DMEM/William's E/UW, kept on ice to maintain tissue integrity during transport (Figure 1). Appropriate ethical approval from the relevant institutional regulatory bodies (IRB) is required, and informed consent may be needed before sampling and storage of human tissue.
b) *Washing the tissue*: Rinse the tissue several times with ice‐cold PBS to remove blood and other contaminants. Ensure that no residual blood remains, as this may interfere with the subsequent enzymatic cell digestion.
c) *Mincing the tissue*: Use sterile scalpels or scissors, finely mince the tissue into small pieces (e.g., 1‐2 mm). Thoroughly mince the tissue but avoid damaging the cells.
d) *Mechanical dissociation*: Mechanically dissociate the tissue by pipetting it “up and down” several times in the tube using a 10 mL strip pipette. This step dissolves cell agglomerates, facilitating a single‐cell suspension.
e) *Enzymatic digestion*: Prepare the enzyme digestion solution by combining collagenase IV (e.g., Gibco 17104019, 0.2‐2.5 mg/mL) and Dispase II (e.g., Gibco 17105041, 1 mg/mL) and or DNase I (e.g., Sigma‐Aldrich DN25, 0.1 mg/mL) in DMEM. Place the minced tissue in the enzyme solution and incubate at 37°C for 30‐60 min, shaking gently to promote digestion (Temperature‐controlled shaker/GentleMACSTM Dissociator). This step allows the breakdown of extracellular matrix and facilitates the release of individual cells.
f) *Filtering*: Pass the digested tissue suspension through a 70 µm cell strainer to remove undigested fragments. This ensures that only single cells pass through for further processing.
g) *Centrifugation*: Centrifuge at 300×*g* for 5‐10 min at 4‐8°C (e.g., Beckman Coulter Avanti J‐15R). After centrifugation, discard the supernatant and resuspend the cell pellet in fresh cold PBS with 0.5‐1% albumin (human/bovine).
h) *Cell counting*: Count the number of cells using a microscope, hemocytometer, or automated cell counter.

*Additional notes*: Maintain sterile conditions throughout the procedure to avoid contamination.

The choice of enzymatic and mechanical dissociation methods must balance efficient matrix degradation with the preservation of cell viability and surface markers. Collagenase type IV is widely used for soft parenchymal organs such as the liver and pancreas due to its moderate proteolytic activity and ability to efficiently degrade collagen rich extracellular matrix without excessive damage to epithelial or stem cells [24, 105]. Dispase II, which cleaves fibronectin and collagen IV, is often combined with collagenase to improve tissue dissociation while maintaining intercellular junction integrity, particularly for epithelial organoids [68, 75]. DNase I is included to prevent DNA induced cell clumping following tissue disruption, enhancing single cell yield and suspension homogeneity [106].

The duration and temperature of enzymatic digestion are critical determinants of cell recovery and quality. Short digestion times (30‐45 min at 37°C) generally preserve viability for delicate tissues such as hepatic or intestinal biopsies, whereas denser tissues like kidney or tumor samples may require up to 60 min of incubation with gentle agitation [107, 108].Over digestion can lead to cell death and loss of stem cell markers such as Lgr5 and EpCAM, while under digestion results in incomplete dissociation and poor plating efficiency. Mechanical trituration by pipetting is therefore performed in parallel to minimize enzymatic exposure while achieving uniform single cell suspensions [24, 68, 109].

In summary, the combination of controlled enzymatic digestion and gentle mechanical dissociation maximizes single cell recovery while maintaining organoid forming potential. Enzyme activity and exposure time should always be empirically optimized for each tissue type and sample size.

### 3.2 Organoid Culture Initiation

*Plating*: Isolated cells are resuspended in Matrigel and seeded as 10‐33 µL domes in prewarmed 12/24‐well plates. Such plates are incubated at 37°C for 10–15 min to solidify.

*Culture medium*: Once the cell domes solidify, overlay with advanced DMEM/F12 medium supplemented with essential growth factors and inhibitors to support initial organoid formation (Tables 2 and 3) and maintain the cultures in a 37°C incubator with 5% CO_2_.

### 3.3 Maintenance and Expansion

a) *Medium change*: Change the medium every 2‐3 days. Carefully remove old media using aspirator or pipette. And carefully add fresh organoid media form the side of the well, to prevent interfering and lifting of cell domes.
b) *Passage*: Passage organoids every 7‐10 days based on morphologies and growth rate, by mechanical disruption using a pipette or digestion with TrypLE Express (10 min at 37°C). Transfer organoids to 15 mL Falcon tubes, wash the organoid with additional advanced DMEM F12 media. This step is followed by a centrifugation (300×*g* for 5 min), resuspension, and re‐embedding in fresh Matrigel, that was cultured with fresh medium containing growth factors as described (Tables 2 and 3).

*Note*: Organoids are typically expanded through at least three passages before being used in downstream experiments to ensure stability and reproducibility.

### 3.4 Differentiation and Maturation

For ES/iPSC differentiation into specific cell lines a lineage‐specific differentiation medium must be used. The results can be confirmed by qPCR analysis to assess lineage‐specific gene expression. Mass spectrometry can be used for metabolic and protein profiling and immunostaining to visualize lineage‐specific markers. The generation of cholangiocyte‐like cells from iPSC relies on FGF10 and activin TGF‐β signaling activators [110, 111] as well as RA, which further enhances cholangiocyte differentiation [54, 64].

#### 3.4.1 Markers

To confirm the culture of specific cell types, both fluorescence‐activated cell sorting (FACS) and magnetic‐activated cell sorting (MACS) are widely used for cell isolation and characterization. Immunohistochemical and fluorescence staining and RT‐qPCR can also be employed for phenotypic validation.

For hepatocytes, key markers include albumin, a major liver‐specific protein, hepatocyte nuclear factor 4 alpha (HNF4α), which is essential for hepatic differentiation and function, cytochrome p450 3A4 (CYP3A4), a marker of metabolic activity and drug metabolism capacity, and multidrug resistance‐associated protein 2 (MRP2), a transporter involved in hepatic bile secretion [89].

Cholangiocytes can be identified by the expression of cytokeratin 19 (CK19), cytokeratin 7 (CK7), and γ‐glutamyltransferase markers of biliary epithelial cells [112], EpCAM enriched epithelial cell population, and Sox9 a transcription factor essential for cholangiocyte lineage. Additionally, CD133 positivity is commonly utilized to enrich cholangiocyte populations [31].

For intestinal stem cells, Lgr5, a definitive intestinal stem cell marker, and ephrin type‐B receptor 2 (EphB2) involved in intestinal crypt organization and differentiation are frequently used [67].

Pancreatic progenitor cells are identified using markers such as PDX1, which is critical for pancreatic lineage specification. SOX9, a transcription factor regulating pancreatic progenitor maintenance, and carbohydrate antigen 19‐9 a pancreatic ductal epithelial marker, along with CD133 for pancreatic progenitor cells facilitating their isolation [113].

To study nephron progenitor cells, markers include Wilms Tumor 1, a key regulator of kidney development, paired box gene2, which is essential for nephron differentiation, and SIX homebox2, which maintains nephron progenitor pools during development. CD133 or CD24 staining can be for used for progenitor cell enrichment and sorting [74].

#### 3.4.2 Functional Evaluation

Beyond the identification of lineage‐specific markers, functional validation remains a critical benchmark for assessing the physiological relevance of organoids. Mature hepatocyte organoids are characterized by their ability to perform key hepatic functions, including albumin secretion, urea synthesis, and CYP‐dependent drug metabolism. For instance, studies have reported the recovery of adult primary human hepatocyte (PHH) morphology and enhanced functional markers in organoid‐derived systems, demonstrating a balance between lipid metabolism and proliferation that can be modulated by IL6 or FXR signaling [114]. Similarly, CRISPR‐based functional screening in steatosis models has validated organoid responsiveness to lipogenic pathway regulation and identified the role of *FADS2* in lipid flux modulation [46].

Polarized hepatocyte organoids with canalicular‐like networks have also been established, exhibiting over 40% PHH‐like cells and demonstrating CYP activity and albumin secretion [115, 116, 117]. In addition, the disappearance of fetal markers such as α‐fetoprotein, along with the appearance of adult‐phase metabolic enzymes (UGT1A1, ADH), further confirms hepatic maturation [118, 119, 120]. These findings highlight that hepatocyte organoids not only express canonical identity markers (ALB, HNF4A) but also recapitulate liver‐specific metabolic and detoxification functions.

For cholangiocyte organoids, functional readouts often center on transport capacity, epithelial polarity, and ductal architecture. Microengineered biliary organoid‐on‐chip systems have shown maintained transport activities, primary cilia formation, and glycocalyx integrity under perfusion, mimicking physiological bile flow [121]. Enhanced bile acid transporter gene expression and active bile transport have also been demonstrated in advanced culture systems integrating oxygen permeable scaffolds and microfluidics [116].

Multiomics profiling approaches, including single‐cell RNA sequencing and ATAC‐seq, have further linked functional maturity to specific transcriptional and regulatory networks [117, 122]. These datasets reveal that organoid metabolic competence, ranging from glucose and lipid metabolism to drug detoxification, is closely tied to structural and compositional fidelity. Taken together, functional validation establishes organoids as physiologically relevant models for drug metabolism, disease modeling, and regenerative medicine, bridging the gap between cellular identity and organ level performance.

### 3.5 Cryopreservation

Organoids are harvested after centrifugation as described above.

*Cryopreservation*: Resuspend organoids in CELLBANKER 2/CryoStor CS10 or other freezing medium with a concentration of 1‐2 × 10^6^ cells/mL or 50‐200 organoids/mL.

*Freezing*: Place cryovials in a cell freezing container (Mr Frosty, CoolCell or similar), freeze down in a −80°C freezer. For long‐term storage, transfer to a liquid nitrogen storage tank within 24 h.

*Recovery*: Thaw frozen organoids quickly in a 37°C water bath, resuspend in fresh medium, and re‐embed in Matrigel and culture under standard conditions for expansion.

### 3.6 Organoid Staining and Fixation

Organoid staining and fixation are essential techniques for assessing cellular composition, special arrangement, and structural integrity. Two widely used methods, 3D whole mount staining and 2D sectioning (paraffin‐embedding and Cryo embedding) offer complementary advantages [123, 124]. These techniques provide valuable organoid visualization of cellular composition and spatial arrangement, and both enable quantitative analysis [125].Whole‐mount staining and confocal microscopy offer a depth of field up to 200 µm [125], which is helpful for visualizing larger 3D structures. 2D sectioning is advantageous as it reveals detailed cellular morphology throughout the entire section and overcoming challenges posed by light scattering in thicker samples [125]. 2D sections can then be used for hematoxylin and eosin staining, immunohistochemistry, and fluorescence in situ hybridization. Broutier et al. suggest that whole mount staining is more suitable for antibody signal detection, while paraffin sectioning followed by histological staining is preferred for precise cellular morphology delineation [10]. Together these approaches provide complementary insights, ensuring efficient analysis of organoid model in research and clinical applications (Figure 3).

## 4 Differences Among Main Abdominal Organoids

Following the general methodology of organoid derivation, it is essential to recognize that each abdominal organ exhibits variations in their development, cell sources, and functional applications. These differences arise from the embryological origins and require specialized culture of each different organ.

### 4.1 Gut System

The stomach and intestine originate from the posterior foregut and midgut, respectively [17]. Generation of gastric organoids requires iPSCs to be directed toward a posterior foregut fate through BMP inhibitors, FGF, Wnt activators, and RA, followed by high EGF concentrations [17]. In contrast, intestinal organoids, require Wnt [126, 127, 128], and FGF signaling for “posteriorization” to a develop polarized intestinal epithelium with villus like structures and crypt like zones [59, 65]. These organoids contain all major epithelial cell types, including absorptive enterocytes, Paneth cells, Goblet cells, enteroendocrine cells, and Tuft cells [129]. Notably, Lgr5+ crypt stem cells can generate “mini guts” alone [67, 68], that are genetically and phenotypically stable and capable of weekly passage for years [67].

### 4.2 Liver

Liver organoids can be divided in two main types: hepatocytes and cholangiocytes. Hepatocytes derived from iPSC requires FGF and BMP4 signaling, often incorporating mesenchymal stem cells and endothelial cells to generate liver bud like structures [57]. While hepatocytes and cholangiocytes typically have slow turnover rates, approximately one third of mature bile duct cells can initiate clonal liver organoid growth [17]. Cholangiocyte organoids can also be minimally invasively derived from bile, and most originate from extrahepatic rather than intrahepatic bile ducts [130].

### 4.3 Kidney

Kidney organoids represent one of the most complex human organoid systems [131], comprising over 20 specialized cell types [17], and are predominantly derived from iPSCs. They develop from the posterior intermediate mesoderm, generating two key progenitor populations: ureteric epithelium and metanephric mesenchyme [17]. Recent advances have improved tubular functional maturation, glomerular vascularization, and collecting duct formation [71, 132]. Novel protocols involve 7 days of monolayer culture for intermediate mesoderm induction, followed by 18 days of 3D culture [71]. Technical parameters with significant impact on such variations, need to be carefully and precisely controlled [133] when comparing patient and control lines [131].

### 4.4 Pancreas

Pancreatic organoids, primarily derived from aSCs, represent a platform for studying cancer therapies [75], mainly pancreatic ductal adenocarcinoma [134], a deadly malignancy often diagnosed at advanced stages [135, 136, 137, 138]. These organoids are instrumental in investigating tumor progression, genetic mutations, and drug responses. Patient‐derived organoids (PDOs) generated from patient biopsies [75, 139] facilitate personalized medicine [75] through RNA profiling and pharmacotyping [113, 140, 141]. Additionally, pancreatic organoids have shown an unexpected capacity to differentiate into hepatocyte‐like cells under certain conditions [62].

Cell source variations are notable among these organoids: gut and liver organoids can be cultured from both iPSCs and aSCs, while kidney organoids primarily derive from iPSCs, and pancreatic organoids mainly from aSCs [17, 21, 27, 54, 71, 142, 143]. Despite this specific origin, all systems require Wnt signaling activation through factors like R‐spondin, BMP, and CHIR99021 to promote cell growth [17]. For in vivo environment simulation, host–microorganism interactions [27, 102, 144], and lumen fluid dynamics [74], gut and kidney organoids are particularly well suited for organ‐on‐chip applications.

## 5 Advanced Techniques and Applications of Organoids: Bridging Innovation and Real‐World Impact

**FIGURE 2:**
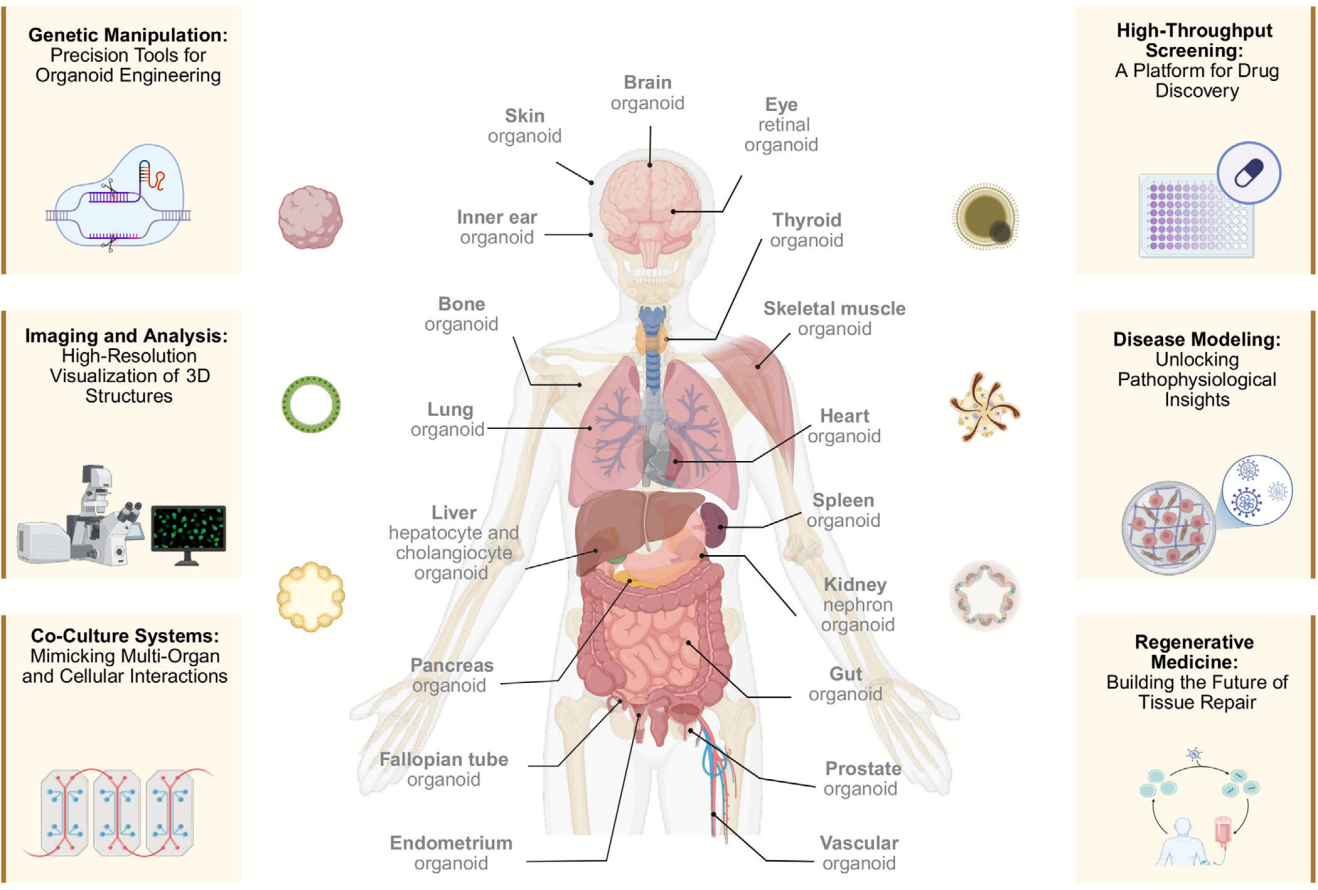
Organoid landscape: a comprehensive platform for research and medicine. This figure shows established organoid models, including brain, skin, eye, thyroid, inner ear, bone, muscle, heart, lung, liver, pancreas, gut, kidney, and more. Key advanced techniques include genetic manipulation (e.g., CRISPR/Cas9), high‐resolution imaging, coculture systems (e.g., organ‐on‐a‐chip), and high‐throughput screening. Applications span disease modeling (e.g., cancer, genetic disorders), drug discovery, and regenerative medicine for tissue repair and organ replacement. Figure was created using BioRender.

Organoid technology has profoundly reshaped modern biomedical research by merging advanced engineering principles with translational applications. This convergence between innovation and practice is refining the study of disease, therapeutic discovery, and the future of regenerative and personalized medicine (Figure 2).

**FIGURE 3:**
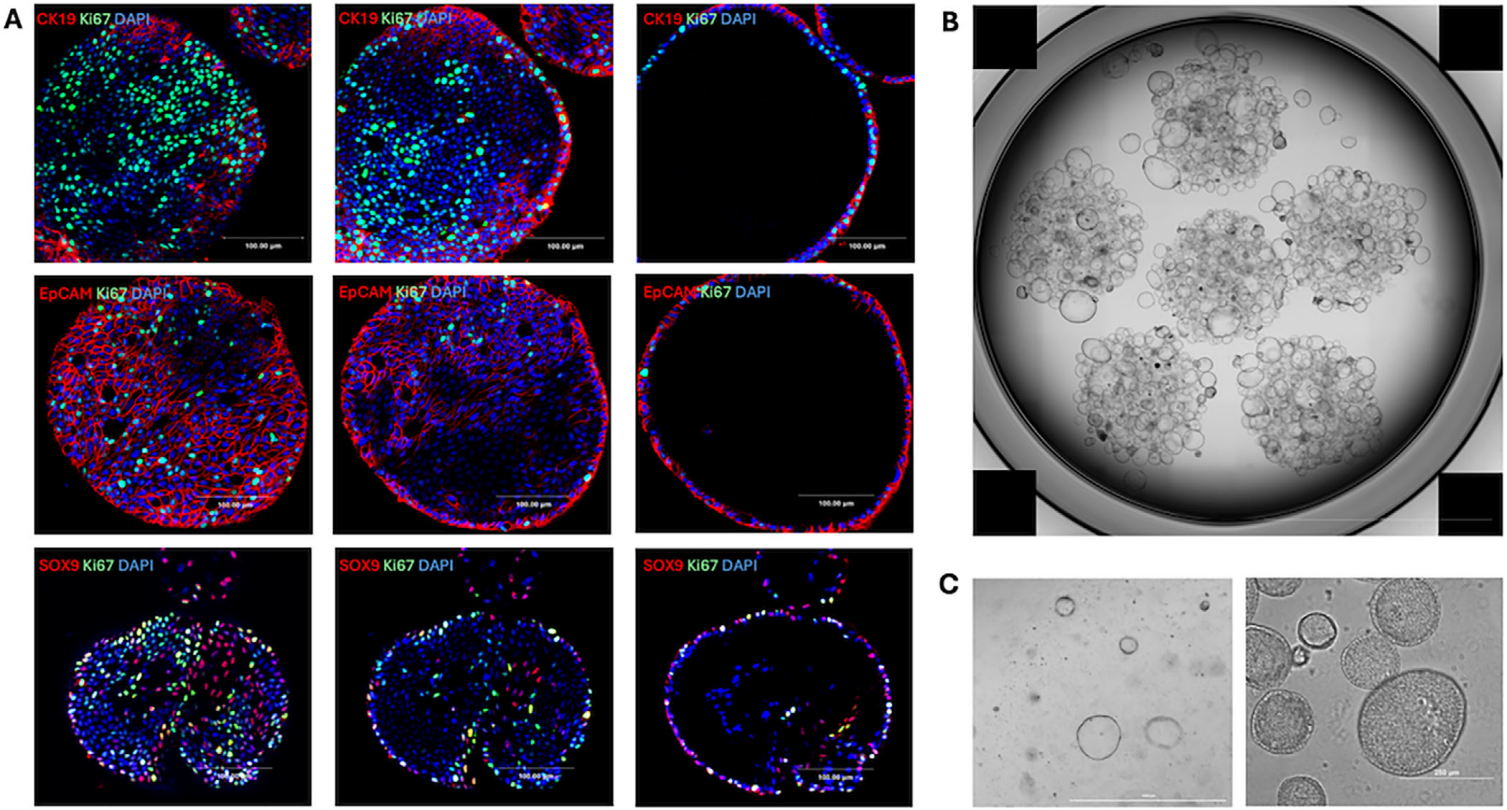
Representative images of cholangiocyte organoids. (A) Immunofluorescence staining of third generation organoids derived from a cholangiocarcinoma patient, demonstrating three different Z‐stack optical sections per row of the same organoid. Organoids are stained with CK19 (red), EpCAM (red), SOX9 (red), Ki67 (green), and DAPI (blue), highlighting the cellular composition, proliferation, and structural organization within different focal planes. Scale bar: 100 µm. (B) Bright‐field image of third generation organoids derived from a discarded donor liver, depicting overall structure and cellular organization. Scale bar: 10,000 µm. (C) Left, bright‐field images of first generation organoids derived from a discarded donor liver after 4 h of cold storage, followed by cryopreservation and isolation. Scale bar: 1000 µm. Right, bright‐field microscopy images showing organoids derived from a cholangiocarcinoma patient. Images illustrate the morphology, structural organization, and heterogeneity of organoids at 20× magnifications. Scale bar: 250 µm. *Abbreviations*: Ki67: marker of proliferation Kiel 67; DAPI: 4′,6‐diamidino‐2‐phenylindole.

### 5.1 Advances in Large‐Scale Organoid Expansion Technologies

The ability to expand organoids at large scale while maintaining physiological relevance is a major frontier in translational biomedical research. Early researchers relied heavily on static culture systems, simple setups involving Matrigel domes in small well plates. Though useful at first, such methods have long struggled with uneven nutrient delivery and diffusion limits. Problem tends to be obvious when cultures are kept for more than weeks.

Over the last few years, dynamic culture systems have been investigated to overcome these limitations. Instead of keeping the organoids still, the new approaches keep them gently moving or perfused, sometimes even watched over by robotic systems. Such as bioreactor‐based culture, microfluidic perfusion systems, and automated liquid‐handling platforms. These approaches differ in design, yet they all aim for one common goal, which is to make organoid growth more reproducible, scalable, and suitable for both basic research and industrial application.

#### 5.1.1 Bioreactor‐Based Expansion

Of all current strategies, the bioreactor remains the most mature and widely used tool for expanding organoids. Its key advantage is simple: the system keeps the culture mixed and oxygenated at all times, reducing the steep nutrient gradients that occur in traditional static setups [145]. Stirred tank, rotating wall, and wave bioreactors have all been applied to support suspension‐based organoid growth under low shear conditions [146]. For instance, Licata et al. demonstrated that rotating wall bioreactors enhanced human intestinal organoid yield 10‐fold while preserving cellular diversity and genomic stability [147]. Sassi et al. achieved sustained proliferation of hepatic organoids over 30 days using a perfused bioreactor [148], and Qian et al. showed that brain organoids can be further maintained over 200 days under controlled bioreactor condition [149].

The benefits of this setup are obvious: precise control of oxygen, nutrients, and pH; lower contamination risks; and the potential for integration with automated systems. Yet, the technology is not without its flaws. Continuous stirring can damage fragile epithelial tissues, especially cholangiocyte‐rich regions. Maintaining sterility at large volumes and calibrating oxygen sensors adds complexity and cost [145]. Still, bioreactors remain the most realistic path toward scalable organoid culture. When combined with soft hydrogel scaffolds or microcarriers to buffer shear forces, they allow researchers to strike a balance between yield and structural fidelity.

#### 5.1.2 Microfluidic Perfusion Systems

If bioreactors solve the “quantity” problem, microfluidic systems address “quality.” These devices, often referred to as organoid‐on‐chip platforms, mimic the perfusion and microcirculation found in real tissues. By maintaining a slow but continuous flow of fresh medium, they ensure that every part of the organoid receives nutrients and oxygen while waste products are washed away [150]. Papamichail et al. observed that liver organoids cultured inside microfluidic chips displayed more pronounced cell polarity, distinct bile canaliculi, and higher metabolic stability than those grown in static wells [151]. Quintard et al. built a chip that supported vascularization around organoids. Endothelial cells formed organized networks that wrapped around the tissue, enhancing perfusion and long‐term survival [152].

What makes these systems unique is their fine control over the environment. Shear stress, oxygen levels, and local chemical gradients can all be tuned in real time, giving researchers the power to direct differentiation or maturation [153]. They also make coculture easier, different cell types can share the same circuit, enabling studies on cross‐organ interactions, inflammation, and drug metabolism [153]. The downsides, however, are mainly practical. Most chips are small, require technical expertise to fabricate, and cannot yet handle industrial‐scale production. Materials such as polydimethylsiloxane (PDMS) may absorb hydrophobic drugs, which complicates pharmacokinetic studies. Future designs may overcome these limits through multichip arrays or by integrating microfluidic precision with bioreactor‐level capacity.

#### 5.1.3 Automated Liquid‐Handling and Robotic Platforms

Automation and robotics are increasingly transforming organoid research into a reproducible, high‐throughput discipline. Automated liquid‐handling systems standardize media exchange, cell seeding, and compound addition across hundreds of culture wells. Novak et al. developed an automated “Interrogator” platform that integrates and maintains up to 10 vascularized Organ Chips for multiorgan coupling and real‐time pharmacokinetic modeling [154].The appeal for pharmaceutical research is obvious: reproducibility, traceability, and higher throughput. Automated platforms provide unparalleled reproducibility, real‐time monitoring, and compatibility with imaging [155]. Yet, as with most automated systems, the technology is expensive and not always flexible. Robotic systems are expensive to install and maintain and may struggle with nonstandard matrices or large volume 3D scaffolds. They excel in discovery‐oriented contexts, such as drug screening and toxicology testing, rather than mass organoid production.

Each of these systems offer distinct benefits, none alone fully satisfies the combined needs of clinical scalability, cost effectiveness, and biological fidelity. Of note, hybrid platforms are emerging that combine perfused bioreactor systems with microfluidic precision and robotic control. For instance, some companies have been developing AI‐monitored hybrid bioreactor enabling adaptive regulation of oxygen and nutrient delivery based on real‐time organoid metabolic profiles. Such approaches may define the next generation of GMP‐compliant organoid manufacturing.

### 5.2 Genetic Manipulation: Precision Tools for Organoid Engineering

Genetic manipulation techniques, such as clustered regularly interspaced short palindromic repeats/CRISPR‐associated protein 9 (CRISPR/Cas9), have become a focal point in organoid research. These tools enable precise genome editing, allowing scientists to model‐specific genetic mutations associated with diseases like cancer and genetic disorders. For instance, the CRISPR–Cas9 and homology‐independent organoid transgenesis (CRISPRHOT) approach, enables efficient gene knock‐in in both human fetal hepatocyte and adult liver ductal organoid systems [123]. Additionally, FatTracer, a CRISPR screening platform designed to identify steatosis modulators and potential therapeutic targets using APOB^−/−^ and MTTP^−/−^ organoids, facilitates research into the etiology of steatosis and drug discovery [46]. This ability to genetically engineer organoids has significantly expanded the potential for studying gene functions and advancing personalized medicine strategies [10].

### 5.3 Imaging and Analysis: High‐Resolution Visualization of 3D Structures

Advanced imaging technologies, including confocal, light‐sheet, live imaging techniques, and two‐photon microscopy, alongside AI, are revolutionizing organoid research by revealing intricate 3D structures and dynamic processes [156]. Live imaging, though challenging due to optical scattering in 3D organoids, is essential for studying real‐time biological phenomena [125]. New modalities like low‐coherence holotomography enable label‐free, long‐term live imaging [157], while detailed protocols support high‐resolution imaging and 3D reconstruction for both, fixed and live samples, bridging in vitro models with potential clinical applications [125, 158].

Next, 4D bioprinting [159], an evolution of 3D bioprinting that integrates time dependent responsiveness and stimuli‐driven morphogenesis is reshaping the landscape of organoid engineering. Unlike static constructs, 4D bioprinted scaffolds utilize smart biomaterials such as shape‐memory polymers and hydrogel composites that dynamically remodel in response to biochemical or mechanical cues. For example, Chen et al. developed a multimaterial 4D bioprinting system capable of producing heterogeneous constructs that adaptively reorganize over time, significantly enhancing vascularization and structural fidelity in hepatic and neural organoids [159]. Similarly, Chadwick et al. created 4D bioprinted glioblastoma organoid arrays that preserved native tumor heterogeneity and enabled high‐throughput, patient‐specific drug screening within days [160]. Beyond oncology, 4D bioprinting has been extended to generate dynamic cardiac and intestinal organoids with controllable tissue curvature and contractility, offering unprecedented insight into developmental biomechanics [161, 162, 163]. These studies collectively demonstrate how temporal material responsiveness enables organoids to more closely replicate living tissue behavior, bridging the gap between static in vitro constructs and more dynamic in vivo physiology.

### 5.4 Coculture Systems: Mimicking Multiorgan and Cellular Interactions

Coculture systems in organoid research integrating multiple cell types together with endothelial cells simulate the complexity of vascular human tissues which more accurately mimics in vivo environments, promoting enhanced tissue function and disease modeling [23, 47, 164, 165, 166]. For example, the coculture of hepato‐biliary‐pancreatic organoids provides a unique platform for studying human development, congenital disorders, drug development, and therapeutic transplantation. Beyond these applications, this approach holds promise for developing interorgan connectivity models for other organ systems derived from stem cell cultures that replicate physiological interaction across multiple tissue types [56, 167]. Additionally, multiple organoid models such as organ‐on‐chip systems provide the capability to analyze multiorgan interactions by integrating a multitude of real‐time sensors. This platform facilitates automated tracking of biophysical and biochemical parameters, enhancing experimental reproducibility and translational relevance [168]. Multiorgan platforms that integrate liver, heart, and lung organoids offer an in vitro framework for evaluating both, therapeutic effectiveness as well as potential side effects of drug candidates, thereby improving preclinical screening strategy [169].

### 5.5 High‐Throughput Screening: A Platform for Drug Discovery

Organoids serve as powerful platforms for high‐throughput drug screening and toxicity testing. Their ability to closely mimic human physiology allows for more accurate predictions of how drugs will perform in the human body, making them superior to traditional cell lines or animal models. Liver organoid‐based toxicity screen generated from 10 different pluripotent stem cell lines measures viability, cholestatic, and/or mitochondrial toxicity with high predictive values for 238 marketed drugs at four different concentrations (sensitivity 88.7%, specificity 88.9%) [170], serving as a potential assay system for liver toxicology studies and drug screening applications. Similarly, cancer organoids [171] from primary patient material enables a range of therapeutic agents in patient‐specific models, to be tested in the resulting organoid cultures, holding great promise for personalized medicine [29].

### 5.6 Disease Modeling: Unlocking Pathophysiological Insights

Organoids have become powerful tools for modeling various diseases, including cancer, genetic disorders, and ischemic injuries. By replicating disease‐specific conditions in vitro, organoids provide a platform to study disease mechanisms at the molecular and cellular levels. For example, tumor‐derived organoids enabled the investigation of cancer progression and treatment resistance, facilitating drug screening and the development of targeted therapies [172, 173]. Additionally, human liver organoids for modeling hepatitis B virus (HBV) infection has facilitated the study of HBV‐mediated transformation and related tumorigenesis [50, 174, 175]. Notably, liver organoids derived from the patients indeed contained α1‐antitrypsin aggregates and presented increased apoptosis, which might contribute to fibrosis and cirrhosis [9]. Extrahepatic cholangiocyte organoids structure and response to ischemia‐reperfusion injury (IRI) are comparable to that found in vivo, providing a suitable model to study IRI of the bile duct in vitro [176]. This ability to replicate disease states in a controlled environment is driving advances in therapeutic development and precision medicine [52]. As advancements in single cell sequencing and spatial transcriptomics continue to evolve, organoid models are poised to uncover previously unrecognized cellular interactions and disease pathways.

### 5.7 Regenerative Medicine: Building the Future of Tissue Repair

The potential of organoids in regenerative medicine is vast, especially for liver, bile duct, and intestinal tissues. Organoids provide a promising source of functional cells for transplantation, tissue regeneration, and disease modeling in regenerative medicine [177]. Yui et al. [178] first exploited the feasibility to apply organoids to repair induced mice acute colitis. The transplantation of mouse [179] and human [180, 181] hepatocyte organoids, generated much more extensive engraftment. And human cholangiocyte organoids have the ability to regenerate and repair bile ducts [21, 22, 182]. Yoshihara et al. [183] generated human islet‐like organoids from iPSC, which provides a promising alternative to cadaveric and device dependent therapies in the treatment of diabetes. The ability to generate functional tissue from organoids opens new avenues for tissue engineering and organ replacement therapies. However, obstacles remain in scalability, vascularization, and immune integration, which must be addressed before organoid‐based therapies reach clinical applications.

### 5.8 Clinical Trial: From the Bench to Clinical Application

Organoid technologies have rapidly advanced from basic in vitro liver models to preclinical validation in animal transplantation and biliary repair studies, demonstrating their potential in regenerative medicine and precision oncology. To date, all registered liver organoid‐related clinical studies utilize PDOs ex vivo, primarily for precision oncology, treatment response prediction, immune tumor microenvironment analysis, and functional drug testing (Table 5). None of the ongoing studies involve direct organoid transplantation into humans, indicating that organoids are currently implemented as platforms to support clinical decisions rather than therapeutic products.

**TABLE 5:** Overview of ongoing clinical studies utilizing patient‐derived liver organoids.

Clinical trial ID	Start date/location	Status	Clinical context	Organoid use
NCT07214649	2026/no location	Not yet recruiting	Refractory biliary leaks after hepatobiliary surgery or liver transplantation	Cell therapy using autologous cholangiocyte organoids to regenerate bile duct and seal leaks
NCT06929845	2024/Italy	Recruiting	Hepatocellular carcinoma	Disease modeling and drug testing
NCT06856252	2021/Italy	Recruiting	NAFLD/NASH	Disease modeling, drug testing and biomarker discovery
NCT06787625	2024/Italy	Recruiting	Colorectal cancer	Study the function of genes targeted by the activity of the NF‐Y/p53 complex
NCT06755762	2024/Taiwan	Recruiting	Head and neck squamous cell carcinoma; metastatic disease	Disease modeling and drug testing (circulating tumor cells‐derived organoids)
NCT06699524	2022/China	Active, not recruiting	Hepatocellular carcinoma	Drug sensitivity testing and treatment response prediction
NCT06355700	2023/Italy	Recruiting	Hepatocellular carcinoma	Disease modeling (tumor‐immune‐microbiota interactions) and drug testing
NCT06077591	2024/Hong Kong	Recruiting	Hepatocellular carcinoma/colorectal cancer	Personalized treatment selection, drug screening for therapy response
NCT05932836	2023/China	Active, not recruiting	Hepatocellular carcinoma	Drug response prediction (organ‐on‐chip testing)
NCT04753996	2021/USA	Recruiting	Primary sclerosing cholangitis (PSC)	Disease modeling
NCT02436564	2015/UK	Unknown	Cholangiocarcinoma, Hepatocellular carcinoma and Pancreatic neoplasms	Disease modeling and drug testing
NCT03307538	2017/Netherlands	Completed	Perihilar cholangiocarcinoma	Radiotherapy response prediction
NCT05720676	2023/Belgium	Unknown	Breast cancer with liver metastasis	Disease modeling (tumor microenvironment analysis) & drug testing

*Data source*: ClinicalTrials.gov.

While these studies provide essential feasibility, safety, and predictive insights, transitioning organoids into fully interventional and regenerative therapies will require breakthroughs in GMP‐compliant ECM systems, engraftment optimization, long‐term safety, and immune compatibility. Continued innovation in these domains is expected to close the translational gap and enable real clinical applications, including bile duct reconstruction, metabolic function restoration, and direct parenchymal regeneration in liver failure.

## 6 Challenges and Future Directions

For broader implementation in both basic research and clinical settings, several technical and biological hurdles need to be addressed during organoid research progresses. These issues not only limit the consistency of current experiments but also shape the direction of what comes next.

### 6.1 Technical Challenges: Reproducibility, Scalability, and Standardization

One of the primary technical challenges in organoid research is reproducibility. Variations in experimental protocols, starting materials, and culture conditions can lead to inconsistent results across laboratories. This issue is compounded by the complex nature of organoid systems, which require precise control over environmental and biological variables. Moreover, scalability remains a key barrier for transitioning organoid technologies from research to clinical applications. Producing organoids in large quantities without losing functional fidelity is essential for drug screening and regenerative medicine. Standardization of culture protocols, medium compositions, and quality control measures is critical to overcome these challenges and ensure the robustness of organoid models across studies. Automation, bioreactors, and closed system cultures are emerging as potential solutions to increase scalability and reproducibility while reducing variability [174, 184].

### 6.2 Biological Challenges: Mimicking Native Tissue Environment and Cellular Diversity

Although organoids closely mimic many aspects of human tissues, there are limitations in replicating the full complexity of the native environment. Organoids often lack key components such as vascularization, neural inputs, and immune system interactions, which can hinder their functionality and long‐term viability [174]. Additionally, the cellular diversity within organoids is often limited compared with actual human tissues, particularly in organs with complex architecture like the liver or pancreas. This restriction impacts the ability to fully replicate disease states or tissue regeneration processes in vitro [56]. Achieving more comprehensive cellular diversity and tissue‐like architecture is essential for advancing organoid applications in disease modeling and therapeutic development.

### 6.3 Future Prospects: Organoid 2.0, Innovations, and Emerging Technologies

Recent advances in Organoid 2.0 technologies have leveraged bioengineering strategies to overcome limitations in scalability, maturation, and physiological relevance of conventional organoid systems. Several key studies have demonstrated complementary approaches integrating organoids with hydrogels, microfluidics, and bioprinting for enhanced functionality and reproducibility. For instance, a hydrogel‐based biliary organoid‐on‐chip model [121] established a perfusable, tubular network that maintained long‐term stability for over 45 days, reproducing the branching geometry of the intrahepatic biliary tree. This platform enabled functional readouts of transport activity, cilia dynamics, and injury responses. Similarly, volumetric bioprinting of organoid laden constructs [185] achieved centimeter scale tissues within 20 s using optically tunable, soft bioresins (<2 kPa), which supported hepatocytic differentiation, albumin secretion, and metabolic enzyme activity.

High‐throughput production has been addressed by 3D microarray bioprinting on pillar plates [186], which reduces organoid volumes by 10‐50 fold while maintaining uniformity (CV 15‐18%) and compatibility with 384 well formats, enabling predictive hepatotoxicity screening. In parallel, oxygen‐permeable PDMS microwells [116] introduced direct oxygenation through honeycomb geometries, enhancing albumin and CYP expression while supporting bile canaliculi formation and mixed hepatic lineages. To eliminate xenogeneic components, microfluidic generation of PEG‐MMP1‐sensitive microcapsules provided continuous, xeno‐free production of vascularized hepatobiliary microtissues with integrated biliary and vascular networks [187]. Finally, ECM‐independent pluripotent stem cell‐derived organoids combined small molecule guidance with self‐organized vascular and Kupffer cell components, producing highly functional liver like constructs with measurable drug metabolism, urea synthesis, and coagulation factor expression [122].

Despite persistent challenges, organoid research holds significant promise through emerging technological integrations. Advanced bioengineering [188] approaches and organ‐on‐chip systems are addressing current limitations, while AI accelerates innovation through multiscale image processing, high‐throughput multiomics analysis, and novel organoid system development [189, 190]. This technological synergy enables comprehensive environmental exposure investigations across lifestyle and pollutant factors at both individual and population levels, “village in a dish” [191], with integrated human–animal platforms [192] enhancing zoonotic pathogen assessment and pandemic prevention through sophisticated susceptibility studies.

## 7 Conclusion

Organoid technology has revolutionized biomedical research by offering physiologically relevant, 3D systems that closely emulate the cellular architecture, function, and microenvironment of human tissues. In this review, we have provided a comprehensive and beginner‐oriented roadmap for establishing and maintaining organoid cultures, with particular emphasis on hepatobiliary and other abdominal systems. What we hope to achieve is a kind of bridge between the complicated technical side of organoid culture and the practical side that new researchers face every day. By assembling current methods into clear, standardized steps, we make it easier for people from different scientific backgrounds to start organoid experiments in a consistent and reliable way.

Beyond serving as a methodological guide, this review also functions as a conceptual framework for selecting and adapting organoid models to specific research goals. Different organoid platforms, ranging from pluripotent stem cell‐ to adult tissue‐derived models, offer complementary advantages, and the understanding of their technical differences enables informed decision making. As organoid‐based approaches continue to expand, they have become indispensable tools in fields spanning developmental biology, tumor modeling, regenerative medicine, and transplantation research [177, 193, 194, 195, 196, 197]. For example, liver organoids have been used to study hepatocyte maturation and bile duct development [165, 198, 199], tumor organoids have revealed mechanisms of drug resistance and clonal evolution [109, 200], and intestinal or pancreatic organoids have advanced our understanding of host microbe interactions and diabetes pathophysiology [61, 68].

Nevertheless, the rapid diversification of organoid related technologies, such as organ‐on‐chip systems [201], bioprinting [202, 203], and multiorganoid [167], presents both opportunity and challenge. The accelerating innovation often leads to what could be termed a “technological paradox”: as we accumulate increasingly sophisticated models, researchers risk prioritizing technical novelty over biological relevance. It is crucial, therefore, to align model complexity with the scientific question at hand. Excessive pursuit of advanced systems can obscure the clarity of experimental objectives, while simpler models may sometimes offer more direct and interpretable insights. The key lies in determining which model, whether a minimalistic 3D organoid or an advanced multilineage coculture, is most appropriate for answering a given biological question.

Looking forward, organoid technology will continue to evolve along with bioengineering, computational modeling, and high‐content analytical tools. Integrating organoids with single‐cell multiomics [204, 205, 206], CRISPR‐based perturbation screens [207, 208], and spatial transcriptomics [209, 210] will enable deeper insights into cell to cell communication and tissue organization. Advances in scalable production [211, 212], synthetic extracellular matrices [213, 214], and perfused bioreactor systems [215] will pave the way for clinical grade manufacturing and transplantation ready constructs. At the same time, ethical, regulatory, and standardization frameworks must evolve to ensure reproducibility, traceability, and equitable access to organoid technologies.

Ultimately, organoids are not merely “mini organs” in a dish, but powerful conceptual and experimental platforms that redefine how we model disease, personalize therapy, and reimagine regenerative medicine. Whether one is a newcomer researcher in the field or an experienced investigator, the central challenge remains the same: to use this remarkable “biological toy” wisely, to test ideas, to ask specific metabolic questions, and to identify the previously undetected mechanisms of human biology.

## Author Contributions

C. Jiao, O. F. Karakaya, and A. Schlegel contributed to the conception and design of the study and the writing of the manuscript. C. Jiao, S. F. Gonzales, Z. Massoud, N. Dadgar, and N. Leipzig were responsible for the protocol part. O. F. Karakaya, C. Jiao, A. Schlegel, and N. Dadgar contributed to the preparation of the tables. C. Jiao, O. F. Karakaya, N. Dadgar, S. F. Gonzales, and A. Schlegel were involved in the creation of the figures. N. Dadgar, S. F. Gonzales, C. J. Wehrle, H. Hong, R. L. Fairchild, F. Aucejo, W. W. Maa, and J. Melenhorst provided critical revisions and intellectual content for the manuscript. A. Schlegel coordinated all collaborations and provided overall guidance and supervision of the manuscript. All authors have read and approved the final version of the manuscript.

## Funding

This work received funding support from Cleveland Clinic transplant research fund.

## Conflicts of Interest

A.S. is a consultant at Bridge to life ltd and Organox ltd. Both companies are not involved in organoid research or offer related products. All the other authors declare no conflicts of interest.

## Ethics Statement

This review includes representative images derived from patient‐derived liver tissue samples that were obtained and used in accordance with institutional ethical standards and the Declaration of Helsinki. Ethical approval for the collection and use of these human samples was granted by the Institutional Review Board (IRB) of the Cleveland Clinic Foundation under protocol IRB:19‐1550 “Preparation of Liver Tumor Organoids via Miniature 3D Bioprinting for Personalized Patient Treatment.” Informed consent was obtained from all participants or their legal guardians prior to sample collection.

## Data Availability

The authors have nothing to report.
